# Supplementary material for: The cholesterol transport protein GRAMD1C regulates autophagy initiation and mitochondrial bioenergetics
Source: Nat Commun. 2022 Oct 21;13:6283. doi: 10.1038/s41467-022-33933-2 (PMC9586981; doi:10.1038/s41467-022-33933-2)
Supplement: Supplementary file 1 — Supplementary Information [file 41467_2022_33933_MOESM1_ESM.pdf]

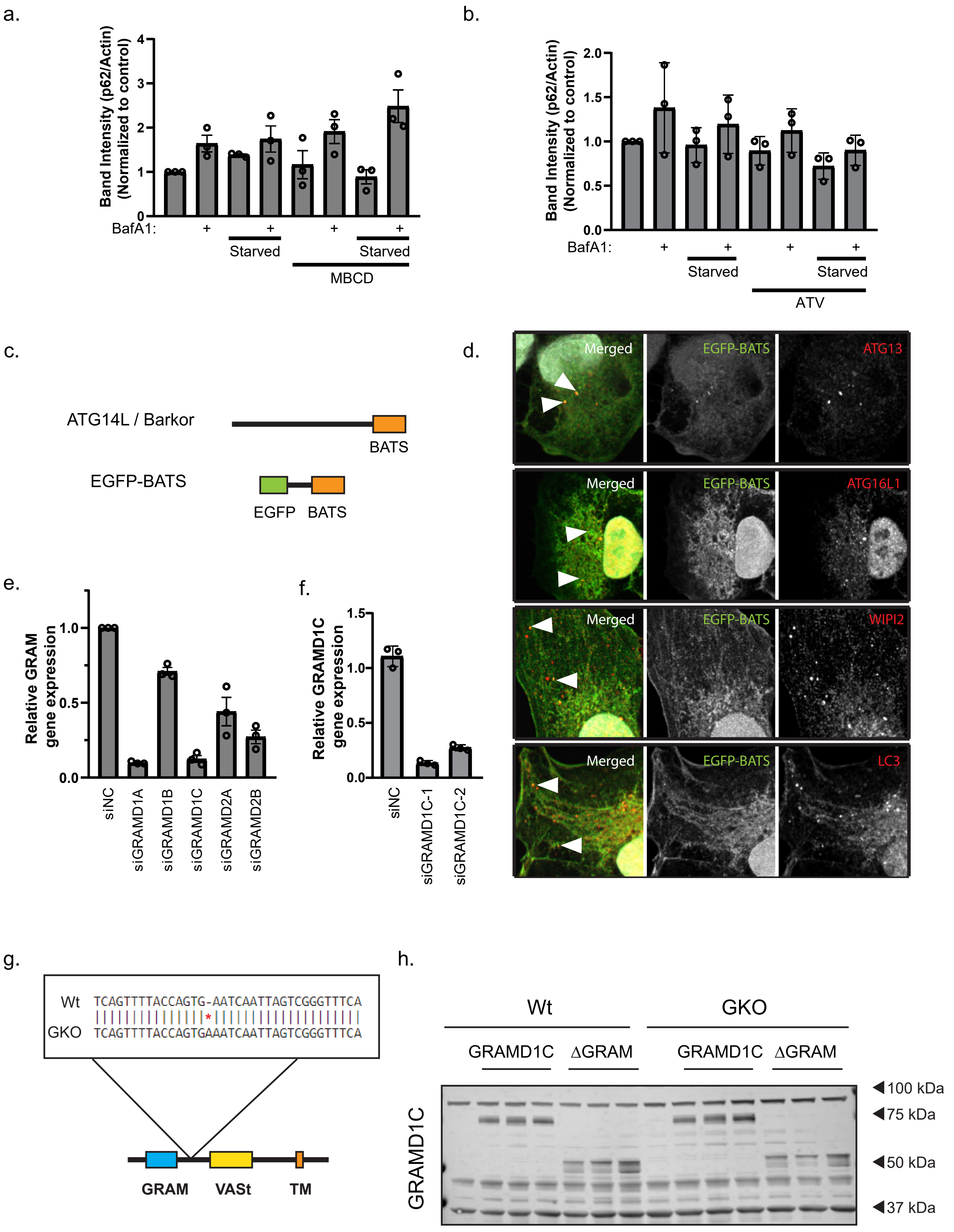

a.
Band Intensity (p62/Actin)
(Normalized to control)
0
1
2
3
4
BafA1:
+
+
+
+
Starved
Starved
MBCD
b.
Band Intensity (p62/Actin)
(Normalized to control)
0.0
0.5
1.0
1.5
2.0
BafA1:
+
+
+
+
Starved
Starved
ATV
c.
ATG14L / Barkor
BATS
EGFP-BATS
EGFP
BATS
d.
Merged
EGFP-BATS
ATG13
Merged
EGFP-BATS
ATG16L1
Merged
EGFP-BATS
WIPI2
Merged
EGFP-BATS
LC3
e.
Relative GRAM
gene expression
0.0
0.5
1.0
siNC
siGRAMD1A
siGRAMD1B
siGRAMD1C
siGRAMD2A
siGRAMD2B
f.
Relative GRAMD1C
gene expression
0.0
0.5
1.0
1.5
siNC
siGRAMD1C-1
siGRAMD1C-2
g.
Wt TCAGTTTTACCAGTG-AATCAATTAGTCGGGTTTCA
GKO TCAGTTTTACCAGTGAAATCAATTAGTCGGGTTTCA
GRAM
VASt
TM
h.
Wt
GKO
GRAMD1C
ΔGRAM
GRAMD1C
ΔGRAM
GRAMD1C
100 kDa
75 kDa
50 kDa
37 kDa

Supplementary figure 1 – EGFP-BATS are recruited to early autophagy structures

a-b The graphs represents the band intensities of p62 relative to actin and normalized to siNC from **a.** Figure 1a and **b.** Figure 1e from n = 3 biologically independent replicates. Error bars = SEM. **c** Graphical representation of ATG14L and its BATS domain, and the EGFP-BATS construct used. **d** U2OS cells stably expressing EGFP-BATS were starved in EBSS for 1 hr prior to fixation and immunostaining with the indicated antibodies prior to imaging with a confocal microscope. Scale bar = 10 µm. n = 3 independent experiments. **e** U2OS cells were treated with siRNA against members of the GRAM family for 72 hrs before mRNA extraction, cDNA synthesis and qPCR using primers against the gene targeted by the siRNA. n = 3 biologically independent replicates. Error bar = SEM **f** U2OS cells were treated with two different siRNAs against GRAMD1C for 72 hrs before mRNA extraction, cDNA synthesis and qPCR using primers against GRAMD1C. n = 3 biologically independent replicates. Error bar = SD. **g** GRAMD1C Knockout Cells (GKO) KO were generated by CRISPR/Cas9, resulting in a single amino acid insertion in exon 7 of *GRAMD1C* leading to a premature stop codon (E245S*fs20). **h** Wt and GKO cells stably expressing untagged GRAMD1C and ΔGRAM were lysed and subjected to western blot analysis for GRAMD1C from 3 experiments. Endogenous GRAMD1C was neither detected in Wt nor GKO cells.

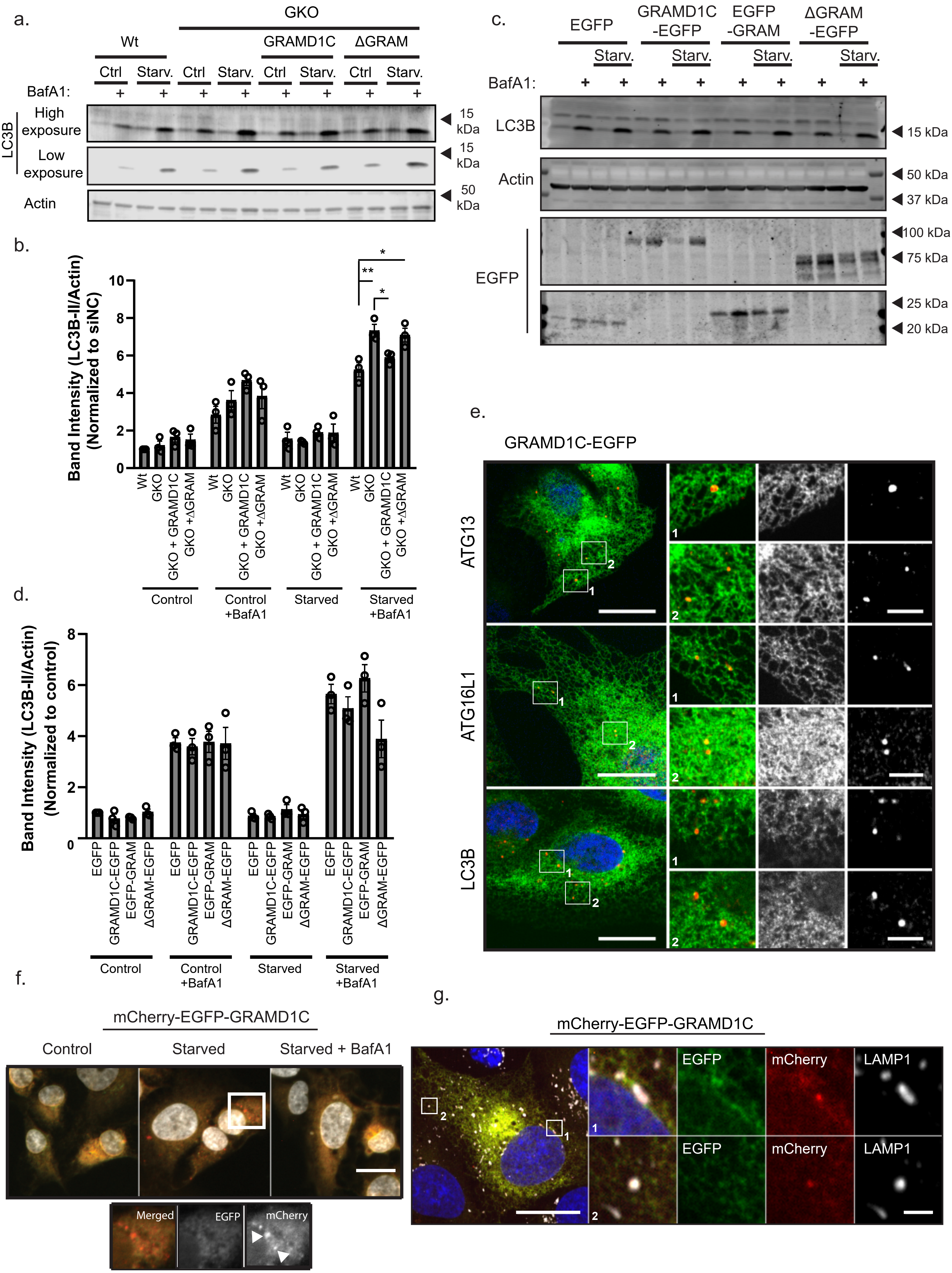
a.
GKO
Wt
GRAMD1C
ΔGRAM
Ctrl
Starv.
BafA1:
LC3B
High exposure
Low exposure
Actin
15 kDa
50 kDa
b.
Band Intensity (LC3B-II/Actin) (Normalized to siNC)
Wt
GKO
GKO + GRAMD1C
GKO +ΔGRAM
Control
Control +BafA1
Starved
Starved +BafA1
c.
EGFP
GRAMD1C -EGFP
EGFP -GRAM
ΔGRAM -EGFP
Starv.
BafA1:
LC3B
Actin
EGFP
15 kDa
50 kDa
37 kDa
100 kDa
75 kDa
25 kDa
20 kDa
d.
Band Intensity (LC3B-II/Actin) (Normalized to control)
EGFP
GRAMD1C-EGFP
EGFP-GRAM
ΔGRAM-EGFP
Control
Control +BafA1
Starved
Starved +BafA1
e.
GRAMD1C-EGFP
ATG13
ATG16L1
LC3B
f.
mCherry-EGFP-GRAMD1C
Control
Starved
Starved + BafA1
Merged
EGFP
mCherry
g.
mCherry-EGFP-GRAMD1C
EGFP
mCherry
LAMP1

Supplementary figure 2 – GRAMD1C knockdown validation and degradation

a Wt cells, GKO cells and GKO cells stably expressing GRAMD1C or GRAMD1C(ΔGRAM) were starved or not in EBSS (± BafA1) for 2 hrs prior to protein isolation and western blot analysis for the indicated proteins. **b** Quantification of LC3-II/Actin band intensity from a. from 3 independent experiments. Significance was determined using 2-way ANOVA followed by Dunnett's multiple comparison test from n = 3 experiments. Error bar = SEM. p value = 0.0012, 0.0044, and 0.0358. **c** U2OS cells expressing EGFP, GRAMD1C-EGFP, EGFP-GRAM, or ΔGRAM-EGFP were incubated in DMEM or EBSS ± 100 nM BafA1 for 4 hrs prior to protein isolation and western blot analysis for the indicated proteins. **d** Quantification of LC3B-II/Actin band intensity from c. from n = 3 replicates. Error bar = SEM **e** U2OS cells stably expressing GRAMD1C-EGFP were starved for 1 hr before fixation and immunostaining for ATG13, ATG16L1 and LC3B. Scale bar = 20 µm, Scale bar inset = 5 µm. An average of 10 cells were imaged per condition. **f** U2OS cells stably expressing mCherry-EGFP-GRAMD1C were starved or not in EBSS (± BafA1) for 4 hrs, prior to fixation and widefield microscopy. Scale bar = 20 µm. Arrows point towards red-only puncta. **g** U2OS cells stably expressing mCherry-EGFP-GRAMD1C were starved for 2 hrs prior to fixation and immunostaining for LAMP1. Scale bar = 20 µm, Scale bar inset = 2 µm.

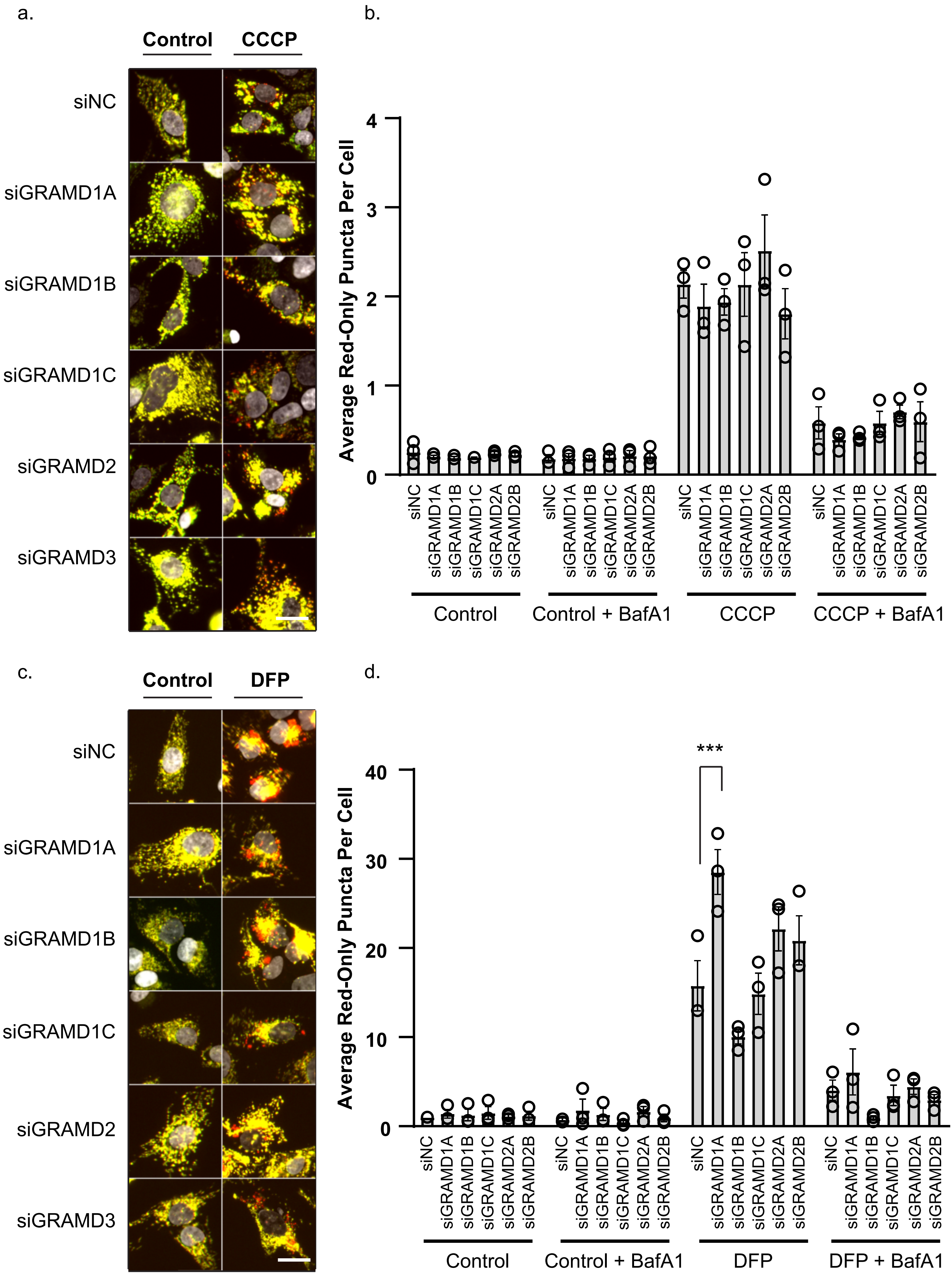
a.
Control
CCCP
siNC
siGRAMD1A
siGRAMD1B
siGRAMD1C
siGRAMD2
siGRAMD3
b.
Average Red-Only Puncta Per Cell
0
1
2
3
4
siNC
siGRAMD1A
siGRAMD1B
siGRAMD1C
siGRAMD2A
siGRAMD2B
Control
Control + BafA1
CCCP
CCCP + BafA1
c.
Control
DFP
siNC
siGRAMD1A
siGRAMD1B
siGRAMD1C
siGRAMD2
siGRAMD3
d.
***
Average Red-Only Puncta Per Cell
0
10
20
30
40
siNC
siGRAMD1A
siGRAMD1B
siGRAMD1C
siGRAMD2A
siGRAMD2B
Control
Control + BafA1
DFP
DFP + BafA1

Supplementary figure 3 - GRAMD1C depletion on PARKIN-dependent/independent mitophagy

a U2OS cells stably expressing MLS (mitochondria localization signal)-mCherry-EGFP were treated with the indicated siRNA for 48 hrs before treatment with 1 mM DFP (+/- 100 nM BafA1) for 24 hrs. The cells were then fixed, and immediately imaged on a high throughput widefield microscope. Scale bar = 20 µM. **b** Quantification of images as shown in (**a**) from n = 3 experiments. The bars represent the average number of red only puncta per cell. Significance was determined using 2-way ANOVA followed by Tukey's comparison test. Error bar = SEM. **c** U2OS cells stably expressing MLS-mCherry-EGFP and untagged Parkin were treated with the indicated siRNA for 72 hrs before treatment with 10 µM CCCP (+/- 100 nM BafA1) for 8 hrs. The cells were then fixed, and immediately imaged on a high throughput widefield microscope. Scale bar = 20 µM. **d** Quantification of images as shown in c from n = 3 experiments. The bars represent the average number of red only puncta per cell. Significance was determined using 2-way ANOVA followed by Tukey's comparison test. Error bar = SEM. p value < 0.0001.

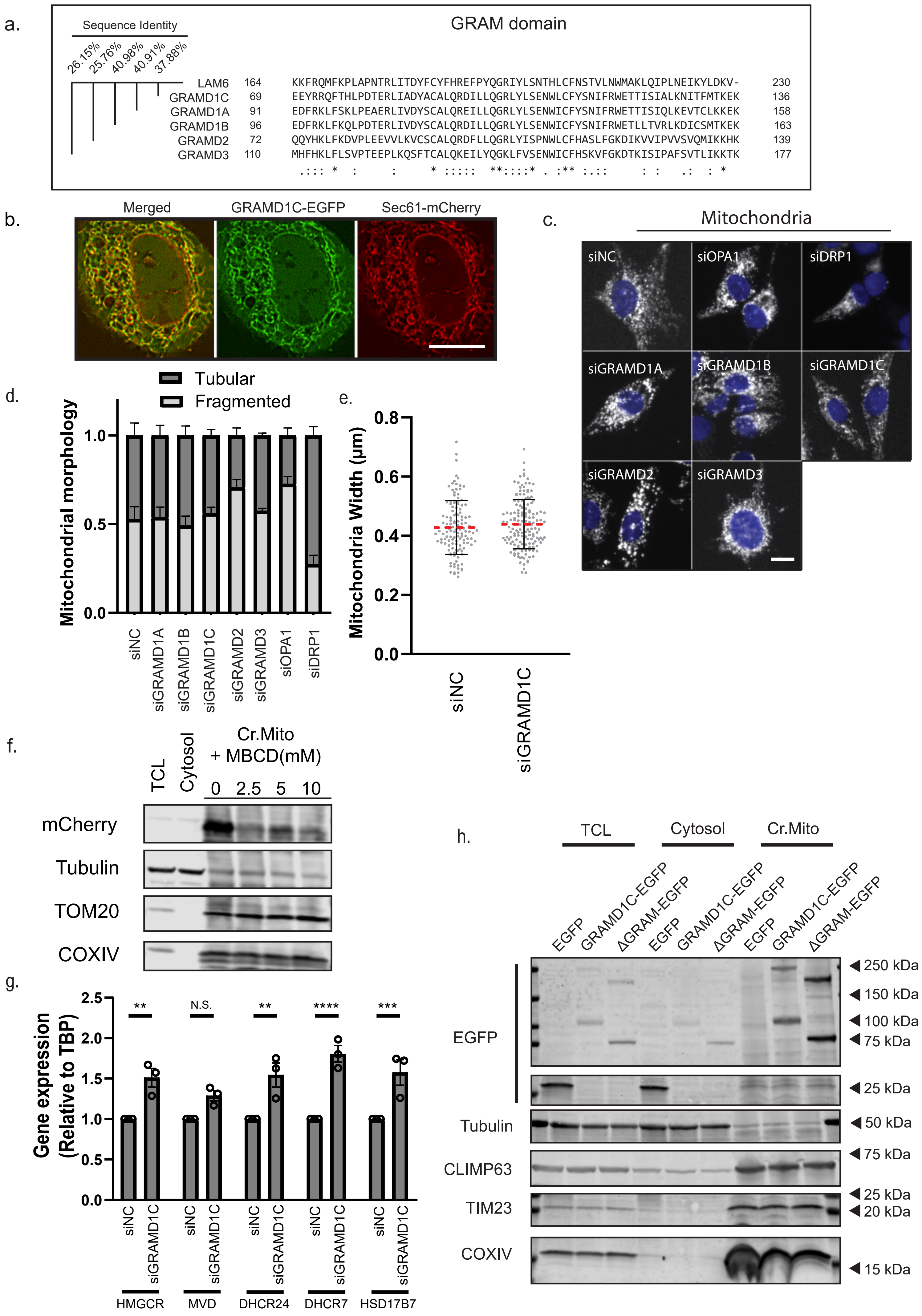
a.
GRAM domain
Sequence Identity
26.15%
25.76%
40.98%
40.91%
37.88%
LAM6 164 KKFRQMFKPLAPNTRLITDYFCYFHREFPYQGRIYLSNTHLCFNSTVLNWMAKLQIPLNEIKYLDKV- 230
GRAMD1C 69 EEYRRQFTHLPDTERLIADYACALQRDILLQGRLYLSENWLCFYSNIFRWETTISIALKNITFMTKEK 136
GRAMD1A 91 EDFRKLFSKLPEAERLIVDYSCALQREILLQGRLYLSENWICFYSNIFRWETTISIQLKEVTCLKKEK 158
GRAMD1B 96 EDFRKLFKQLPDTERLIVDYSCALQRDILLQGRLYLSENWICFYSNIFRWETLLTVRLKDICSMTKEK 163
GRAMD2 72 QQYHKLFKDVPLEEVVLKVCSCALQRDFLLQGRLYISPNWLCFHASLFGKDIKVVIPVVSVQMIKKHK 139
GRAMD3 110 MHFHKLFLSVPTEEPLKQSFTCALQKEILYQGKLFVSENWICFHSKVFGKDTKISIPAFSVTLIKKTK 177
b.
Merged
GRAMD1C-EGFP
Sec61-mCherry
c.
Mitochondria
siNC
siOPA1
siDRP1
siGRAMD1A
siGRAMD1B
siGRAMD1C
siGRAMD2
siGRAMD3
d.
Tubular
Fragmented
Mitochondrial morphology
1.0
0.5
0.0
siNC
siGRAMD1A
siGRAMD1B
siGRAMD1C
siGRAMD2
siGRAMD3
siOPA1
siDRP1
e.
Mitochondria Width (µm)
0.8
0.6
0.4
0.2
0.0
siNC
siGRAMD1C
f.
TCL
Cytosol
Cr.Mito
+ MBCD(mM)
0
2.5
5
10
mCherry
Tubulin
TOM20
COXIV
g.
Gene expression
(Relative to TBP)
2.5
2.0
1.5
1.0
0.5
0.0
**
N.S.
**
****
***
siNC
siGRAMD1C
HMGCR
MVD
DHCR24
DHCR7
HSD17B7
h.
TCL
Cytosol
Cr.Mito
EGFP
GRAMD1C-EGFP
ΔGRAM-EGFP
EGFP
Tubulin
CLIMP63
TIM23
COXIV
250 kDa
150 kDa
100 kDa
75 kDa
25 kDa
50 kDa
75 kDa
25 kDa
20 kDa
15 kDa

Supplementary figure 4 - GRAMD1C depletion leads to increased SREBP target gene expression

a GRAM domain sequence alignment of human *GRAMD1A*, *GRAMD1B*, *GRAMD1C*, *GRAMD2*, *GRAMD3* versus yeast *LAM6* using Clustal Omega. Sequence similarity values were obtained using BlastP. "." represents a conservation of weakly similar properties, ":" represents a conservation of strongly similar properties, "*" represents conserved amino acids. **b** U2OS cells expressing GRAMD1C-EGFP and SEC61-mCherry were fixed and subjected to confocal microscopy analysis. Scale bar = 20 µm. An average of 10 cells were imaged. **c-d** Mitochondrial morphology was characterized in cells expressing MLS-EGFP-mCherry. Mitochondrial EGFP signal was analyzed with CellProfiler Analyst based on the mitochondrial area, texture, intensity distribution, intensity, and shape. Mitochondria from siDRP1 and siOPA1 treated cells were positive controls for tubular and fragmented mitochondria respectively. 3 independent experiments were carried out. Error bar = SD. Scale bar = 20 µm. **e** Mitochondria width measured from electron microscopy images (Figure 6d) from 25 siNC treated cells and 31 siGRAMD1C treated cells. The width of 5 mitochondria were measured per cell. **f** Isolated crude mitochondria were incubated with recombinant mCherry-D4 in the presence of MBCD at 37 ^{o}c for 45 mins prior to western blot analysis. **g** U2OS cells were treated with siRNA against control (siNC) or GRAMD1C before RNA extraction and cDNA synthesis. The graph represents the gene expression of the indicated SREBP target genes relative to the expression of TBP and normalized to siNC from n = 3 independent experiments. Significance was determined using One way ANOVA followed by Sidak's multiple comparison test. Error bar = SEM. p value = 0.0023, 0.0012, <0.0001 and 0.0007. **h** U2OS cells expressing EGFP, GRAM1C-EGFP or ΔGRAM-EGFP were subjected to crude mitochondrial fractionation (Cr.Mito) followed by western blot analysis for the indicated proteins. TLC: total cell lysate.

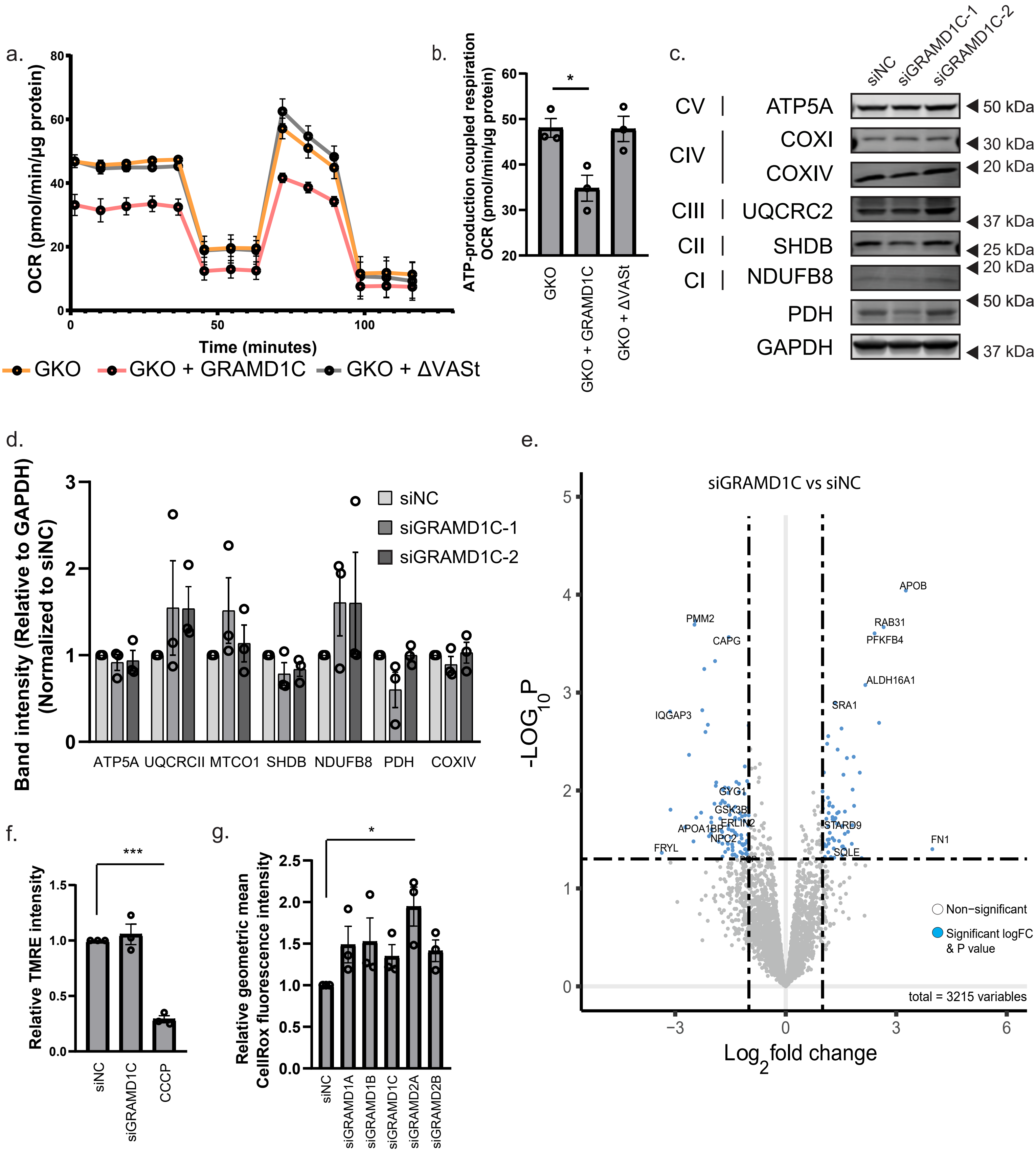

a.
OCR (pmol/min/µg protein)
Time (minutes)
GKO
GKO + GRAMD1C
GKO + ΔVASt
b.
ATP-production coupled respiration OCR (pmol/min/µg protein)
GKO
GKO + GRAMD1C
GKO + ΔVASt
c.
siNC
siGRAMD1C-1
siGRAMD1C-2
CV
ATP5A
CIV
COXI
COXIV
CIII
UQCRC2
CII
SHDB
CI
NDUFB8
PDH
GAPDH
50 kDa
30 kDa
20 kDa
37 kDa
25 kDa
20 kDa
50 kDa
37 kDa
d.
Band intensity (Relative to GAPDH) (Normalized to siNC)
siNC
siGRAMD1C-1
siGRAMD1C-2
ATP5A
UQCRCII
MTCO1
SHDB
NDUFB8
PDH
COXIV
e.
siGRAMD1C vs siNC
$-LOG_{10}P$
Log_2fold change
APOB
PMM2
RAB31
CAPG
PFKFB4
ALDH16A1
SRA1
IQGAP3
GYG1
GSK3B
ERLIN2
APOA1BP
STARD9
NPC2
FN1
FRYL
SQLE
Non−significant
Significant logFC & P value
total = 3215 variables
f.
Relative TMRE intensity
siNC
siGRAMD1C
CCCP
g.
Relative geometric mean CellRox fluorescence intensity
siNC
siGRAMD1A
siGRAMD1B
siGRAMD1C
siGRAMD2A
siGRAMD2B

Supplementary figure 5 – GRAMD1C depletion does not alter mitochondrial morphology and proteins

a Mitochondrial oxygen consumption rate (OCR) was analyzed in GKO, GKO + GRAMD1C and GKO + ΔVASt cells using the Seahorse analyzer. OCR was measured after gradual addition of Oligomycin, CCCP and Rotenone/Antimycin A. n = 3 independent experiments were carried out. Error bar = SEM. p value = 0.0199. **b** ATP-linked respiration is calculated from the difference between the maximal respiratory capacity and the proton leak. Significance was determined using 1-way ANOVA followed by Bonferroni's comparison test from n = 3 experiments. Error bar = SEM. **c** Cell lysates from siNC and siGRAMD1C treated cells were subjected to western blot analysis with antibodies against the indicated OXPHOS components. **d** The graph represents the quantification of band intensities of data in a. relative to GAPDH and normalized to siNC. Significance was determined with 1-way ANOVA followed by Tukey's comparison test from n =3 experiments. Error bar = SEM. **e** Lysates from U2OS cells transfected control (siNC) or GRAMD1C siRNA were subjected to mass spectrometry analysis. The graph represents the difference in protein abundance in siGRAMD1C treated cells compared to siNC cells. Blue spots represent proteins that have $p < 0.05$ and a log fold change of > 1.3 or < -1.3. **f** U2OS cells treated with siRNA against control or GRAMD1C for 72 hrs were stained with Tetramethylrhodamine (TMRE) and dye fluorescence intensity was measured by flow cytometry. CCCP treated cells were used as positive control. Significance was determined using 1-way ANOVA followed by Tukey's comparison test. n = 3 experiments. Error bar = SEM. p value = 0.0003 **g** U2OS cells were treated with the indicated siRNA for 72 hrs before staining with the ROS fluorescent dye CellRox. The cells were then imaged using flow cytometry. Significance was determined using 1-way ANOVA followed by Tukey's comparison test from n = 3 experiments. Error bar = SEM. p value = 0.0174.

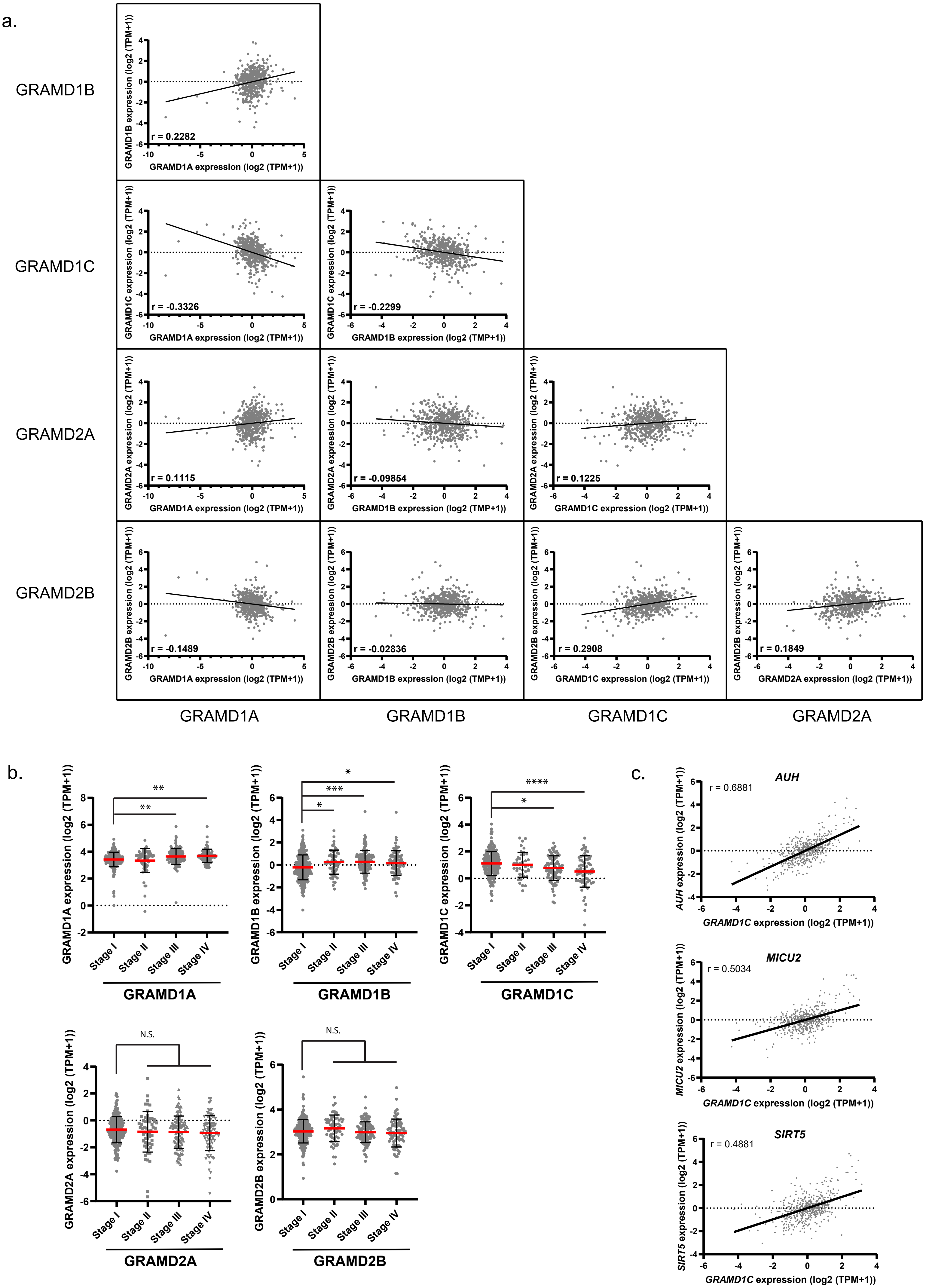
a.
GRAMD1B
GRAMD1C
GRAMD2A
GRAMD2B
GRAMD1A
GRAMD1B
GRAMD1C
GRAMD2A
r = 0.2282
r = -0.3326
r = -0.2299
r = 0.1115
r = -0.09854
r = 0.1225
r = -0.1489
r = -0.02836
r = 0.2908
r = 0.1849
GRAMD1A expression (log2 (TPM+1))
GRAMD1B expression (log2 (TMP+1))
GRAMD1C expression (log2 (TPM+1))
GRAMD2A expression (log2 (TPM+1))
GRAMD1B expression (log2 (TPM+1))
GRAMD1C expression (log2 (TPM+1))
GRAMD2A expression (log2 (TPM+1))
GRAMD2B expression (log2 (TPM+1))
b.
Stage I
Stage II
Stage III
Stage IV
GRAMD1A
GRAMD1B
GRAMD1C
GRAMD2A
GRAMD2B
N.S.
GRAMD1A expression (log2 (TPM+1))
GRAMD1B expression (log2 (TPM+1))
GRAMD1C expression (log2 (TPM+1))
GRAMD2A expression (log2 (TPM+1))
GRAMD2B expression (log2 (TPM+1))
c.
AUH
r = 0.6881
AUH expression (log2 (TPM+1))
MICU2
r = 0.5034
MICU2 expression (log2 (TPM+1))
SIRT5
r = 0.4881
SIRT5 expression (log2 (TPM+1))
GRAMD1C expression (log2 (TPM+1))

Supplementary figure 6 – The expression of GRAMs in ccRCC

a The expression of individual GRAM members from the TCGA-KIRC study were plotted against each other. The Pearson's correlation values are displayed in each graph. **b** The expression of individual GRAMs were plotted against cancer stage in the TCGA KIRC cohort. Significance was determined using 1-way ANOVA followed by Tukey's comparison test. Error bar = SEM. Stage 1 n = 272, Stage 2 n = 59, Stage 3 n = 123 and Stage 4 n = 82. p value (GRAMD1A) = 0.0026 and 0.0015, (GRAMD1B) = 0.0132, 0.0001 and 0.0248, (GRAMD1C) = 0.0191 and < 0.0001. **c** Top three co-expressed mitochondrial genes with *GRAMD1C* in the KIRC TCGA cohort plotted against the expression of GRAMD1C. Top co-expressed genes of *GRAMD1C* in the KIRC TCGA dataset were downloaded from GEPIA2.cancer-pku.cn and subjected to GO Cellular Compartment enrichment using Enrichr[89,90,91].

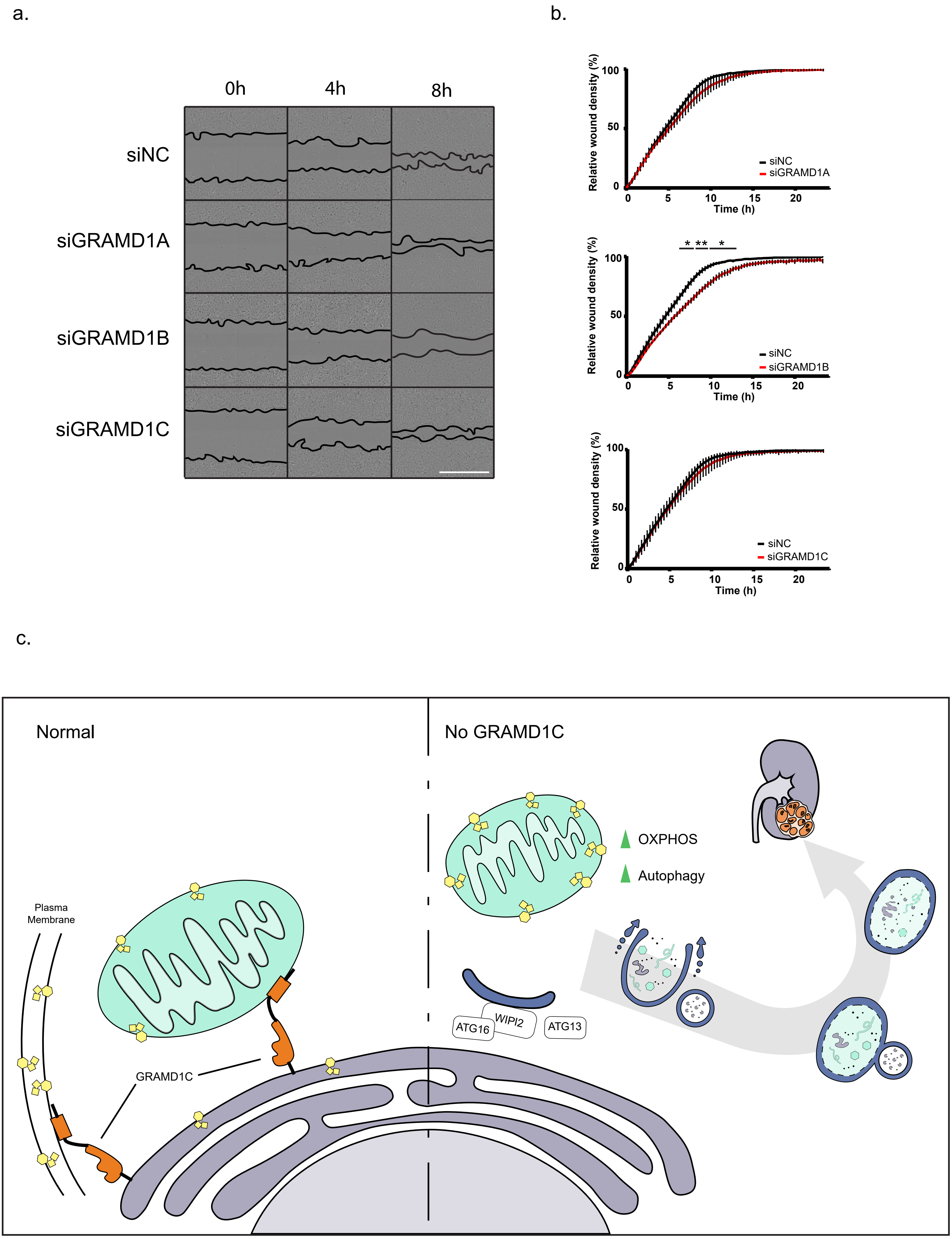

a.
0h
4h
8h
siNC
siGRAMD1A
siGRAMD1B
siGRAMD1C
b.
Relative wound density (%)
100
50
0
0
5
10
15
20
Time (h)
siNC
siGRAMD1A
* ** *
siGRAMD1B
siGRAMD1C
c.
Normal
No GRAMD1C
Plasma Membrane
GRAMD1C
OXPHOS
Autophagy
WIPI2
ATG16
ATG13

Supplementary figure 7 – GRAMD1B regulates cell migration in ccRCC cells

a-b 786-O cells treated with siRNA against GRAMD1A-C were grown to confluency. A rubber stopper was then used to generate a wound, and the cells were immediately imaged at 1 hr intervals using the Incucyte. **a** shows representative images taken at 0, 4 and 8 hrs. **b** The graph represents the relative wound density over time. Significance was determined using 2-way ANOVA from n = 3 individual experiments. Scale bar = 700 µm. Error bar = SD. p value =0.0414, 0.0075 and 0.0202 **c** Graphical summary of the role of GRAMD1C as a negative regulator of autophagy. GRAMD1C localizes to the ER and interacts with mitochondria and the plasma membrane via its GRAM domain to facilitate cholesterol transport from these cellular structures to the ER. Such property of GRAMD1C may contribute to the suppression of autophagosome biogenesis by modulating cholesterol levels at ER membranes that are associated with autophagosome initiation sites. Upon GRAMD1C depletion membrane recruitment of early autophagic markers and autophagic flux are increased, as well as mitochondrial respiration and cholesterol abundance, potentially contributing to the decreased survival of ccRCC patients with low GRAMD1C levels.

Primers used in this study			
**Target**	**Application**	**Forward (5'->3')**	**Reverse (5'->3')**
TBP	qPCR	CAGAAAGTTCATCCTCTGGGCT	TATATTCGGCGTTTCGGGCA
GRAMD1C	qPCR	GTCCTTTTACCGTCTCCGCC	AGTCTCGGAGCACTCCCTTTA
DHCR24	qPCR	CGATGCACTCCGTCCGAAAA	GATGATGCGGATCTCAGCGG
DHCR7	qPCR	ATCGCTGACATCATCCGGGG	TAAAGTCCTGCGCCCACCTTC
HSD17B7	qPCR	CTTCCAGCACAGCAAAGGCA	CCACATTGGAATAGAGACCCTGC
HMGCR	qPCR	GCCCTCAGTTCCAACTCACA	CAAGCTGACGTACCCCTGAC
MVD	qPCR	CGTAAGTGGCTGTGGAGCTG	CGTAAGTGGCTGTGGAGCTG
GRAMD1A	qPCR	AGCGAGAAGCATTTCTTCACTT	CAGTGCATTCTGCCAGAGG
GRAMD1B	qPCR	AGAAGGGCTCAGATCACTCCT	GTGGGGCTTAACACATTATACCA
GRAMD2A	qPCR	TGGCCGAGAAGGGATAACAC	GCCAGTTGGGGGAGATGTAG
GRAMD2B	qPCR	GCCCTTCATCTCTGCCTCTG	GCCCTTCATCTCTGCCTCTG
GRAMD1C	Crispr	CACCGAACCCGACTAATTGATTCAC	AAACGTGAATCAATTAGTCGGGTTC
EGFP-BATS	Gibson	TTTAAACTTAAGCTTGGTACGCCACC ATGGTGAGCAAG	TCCTGAACCTGATCCTGAACCGCTCTTG TACAGCTCGTCCATG
EGFP-BATS	Gibson	AGCGGTTCAGGATCAGGTTCAGGAG CTGGAGAATCAGATGAG	TTTAAACGGGCCCTCTAGACTTAACGGT GTCCAGTGTAAG
GRAMD1C	Gibson	GATCTCGAGCTCAAGCTTCGGCCACC ATGGAGGGCGCT	TCCTGAACCTGATCCTGAACCGCTGCTT TCAACAGCCATGCCAGTCTTATTC
GRAMD1C	Gibson	AGCGGTTCAGGATCAGGTTCAGGAG TGAGCAAGGGCGAGGAG	AGAATTATCTAGAGTCGCGGTTACTTGT ACAGCTCGTCCATG
GRAMD1C-V1-EGFP	Cloning	AGCGGTTCAGGATCAGGTTCAGGAG AGGGCGCTCCGACTGTC	TTTAAACGGGCCCTCTAGACTTACTTGT ACAGCTCGTCCATGC
3xHA-EGFP-OMP25	Cloning	CAAAAAAGTTGGCACCATGTGCCACC ATGTATCCCTATG	TGTACAAGAAAGTTGATTACTCAGAGCT GCTTTCGGTATC
GRAMD1C (ΔGRAM)	Cloning	TTGAGTACTTACGTAGGTACATGGAA AACTTGTCACTGTCG	CTGATCCTGAACCGCTGCTTTCAACAGC CATGCC
GRAMD1C (ΔVASt)	Gibson	GATCTCGAGCTCAAGCTTCGGCCACC ATGGAGGGCGCT	CAATGGCCTGATGAAGATCTTTCTCAGG AACATTTTCTTCATCAACAGAATCTG
GRAMD1C (ΔVASt)	Gibson	AGATCTTCATCAGGCCATTGAAGACC CTG	AGAATTATCTAGAGTCGCGGCTAGCTTT CAACAGCCATGC

Supplementary Table 1. Description of the primers used in this study

Plasmids used in this study		
**Vector**	**Description**	**Source**
pENTR3C	Gateway Entry vector	Invitrogen
pENTR3C-GRAMD1C	Human GRAMD1C PCR product (from U2OS cDNA) was cloned into pENTR3C.	This study
pENTR3C-GRAMD1C (ΔGRAM)	Cloning human GRAMD1C lacking the GRAM domain into a pENTR3C backbone.	This study
pLenti-PGK-GRAMD1C-EGFP	A lentiviral vector expressing C-terminal EGFP tagged GRAMD1C was generated using Gibson Assembly.	This study
pLenti-PGK-GRAMD1C (ΔGRAM)-EGFP	A lentiviral vector expressing C-terminal EGFP tagged GRAMD1C (ΔGRAM) was generated using Gibson Assembly.	This study
pCMV-VSV-G	Envelope protein vector for lentivirus production.	Gift from Bob Weinberg (Addgene # 8454)
psPAX2	Lentiviral packaging vector for lentivirus production.	Gift from Didier Trono (Addgene # 12260)
pLenti-PGK-EGFP-GRAM domain	EGFP-GRAM domain was cloned from an entry vector into a pLenti-PGK vector using Gibson Assembly.	This study
pcDNA5-MLS-EGFP-mCherry	Vector for inducible mitophagy reporter using the FlpIn system.	[37]Reference
pLenti-PGK-3xHA-EGFP-OMP25	3xHA-EGFP-OMP25 vector used for affinity purification of mitochondria. The insert was cloned from [81].	This study
pLenti-PGK-EGFP-BATS	EGFP-BATS domain cloned from ATG14L plasmid into a pLenti-PGK vector using Gibson Assembly.	This study
pDest-FlpIn-mCherry-EGFP-GRAMD1C	Doxycycline inducible mCherry-EGFP-GRAMD1C construct generated using gateway cloning.	This study
pLVX-CMV-Puro-GRAMD1C	Lentiviral vector expressing GRAMD1C was generated using Gibson Assembly.	This study
pLVX-CMV-Puro- GRAMD1C (ΔGRAM)	Lentiviral vector expressing GRAMD1C (ΔGRAM) was generated using Gibson Assembly.	This study
pLVX-CMV-Puro-GRAMD1C (ΔVASt)	Lentiviral vector expressing GRAMD1C (ΔVASt) was generated using Gibson Assembly.	This study

Supplementary Table 2. Description of the plasmids used in this study.
