# Supplementary material for: The cholesterol transport protein GRAMD1C regulates autophagy initiation and mitochondrial bioenergetics
Source: Nat Commun. 2022 Oct 21;13:6283. doi: 10.1038/s41467-022-33933-2 (PMC9586981; doi:10.1038/s41467-022-33933-2)
Supplement: Supplementary file 3 — Description of Additional Supplementary Files [file 41467_2022_33933_MOESM3_ESM.pdf]

Description of Additional Supplementary Files

File name: Supplementary Data 1
Description: Proteins enriched from co-immunoprecipitation of GRAMD1C-EGFP were compared against proteins enriched from co-immunoprecipitation of EGFP-tag alone. The log fold change (LogFC), P-values and protein IDs of the significant proteins are described. The data have been deposited in the PRIDE database under accession code PXD033125 [https://www.ebi.ac.uk/pride/archive/projects/PXD033125].

File name: Supplementary Data 2
Description: Proteins enriched from co-immunoprecipitation of GRAMD1C(ΔGRAM)-EGFP were compared against proteins enriched from co-immunoprecipitation of EGFP-tag alone. The log fold change (LogFC), P-values and protein IDs of the significant proteins are described. The data have been deposited in the PRIDE database under accession code PXD027502 [https://www.ebi.ac.uk/pride/archive/projects/PXD027502].
