# Supplementary material for: The cholesterol transport protein GRAMD1C regulates autophagy initiation and mitochondrial bioenergetics
Source: Nat Commun. 2022 Oct 21;13:6283. doi: 10.1038/s41467-022-33933-2 (PMC9586981; doi:10.1038/s41467-022-33933-2)
Supplement: Supplementary file 6 — Reporting Summary [file 41467_2022_33933_MOESM6_ESM.pdf]

nature portfolio

Corresponding author(s): Matthew Ng Yoke Wui
Anne Simonsen

Last updated by author(s): September 8, 2022

Reporting Summary

Nature Portfolio wishes to improve the reproducibility of the work that we publish. This form provides structure for consistency and transparency in reporting. For further information on Nature Portfolio policies, see our Editorial Policies and the Editorial Policy Checklist.

Statistics

For all statistical analyses, confirm that the following items are present in the figure legend, table legend, main text, or Methods section.

n/a	Confirmed	
☐	☒	The exact sample size (n) for each experimental group/condition, given as a discrete number and unit of measurement
☐	☒	A statement on whether measurements were taken from distinct samples or whether the same sample was measured repeatedly
☐	☒	The statistical test(s) used AND whether they are one- or two-sided<br>*Only common tests should be described solely by name; describe more complex techniques in the Methods section.*
☒	☐	A description of all covariates tested
☒	☐	A description of any assumptions or corrections, such as tests of normality and adjustment for multiple comparisons
☐	☒	A full description of the statistical parameters including central tendency (e.g. means) or other basic estimates (e.g. regression coefficient) AND variation (e.g. standard deviation) or associated estimates of uncertainty (e.g. confidence intervals)
☐	☒	For null hypothesis testing, the test statistic (e.g. F, t, r) with confidence intervals, effect sizes, degrees of freedom and P value noted<br>*Give P values as exact values whenever suitable.*
☒	☐	For Bayesian analysis, information on the choice of priors and Markov chain Monte Carlo settings
☒	☐	For hierarchical and complex designs, identification of the appropriate level for tests and full reporting of outcomes
☒	☐	Estimates of effect sizes (e.g. Cohen's d, Pearson's r), indicating how they were calculated

Our web collection on statistics for biologists contains articles on many of the points above.

Software and code

Policy information about availability of computer code

Data collection	Odyssey Li-cor (3.0), Zen Blue (2.3), FLUOstar OPTIMA (v2.20R2)
Data analysis	CellProfiler software (2.2.0, 3.1.9 and 4.07), CellProfiler Analyst (2.2.1), MaxQuant 1.6.17.0, GraphPad Prism (8.01), Seahorse Analytics software (Wave 2.3), TCGABiolinks R package, Perseus, Limma, Package R

For manuscripts utilizing custom algorithms or software that are central to the research but not yet described in published literature, software must be made available to editors and reviewers. We strongly encourage code deposition in a community repository (e.g. GitHub). See the Nature Portfolio guidelines for submitting code & software for further information.

Data

Policy information about availability of data

All manuscripts must include a data availability statement. This statement should provide the following information, where applicable:

- Accession codes, unique identifiers, or web links for publicly available datasets
- A description of any restrictions on data availability
- For clinical datasets or third party data, please ensure that the statement adheres to our policy

All data is available upon reasonable request. Source data are provided with this paper. The proteomics data generated in this study have been deposited in the PRIDE database under accession code PXD033125 [https://www.ebi.ac.uk/pride/archive/projects/PXD033125] (Supplementary Table 1 data) and PXD027502 [https://www.ebi.ac.uk/pride/archive/projects/PXD027502] (Supplementary Table 2 data). Raw data for Fig 1a-f, h, i, k; Fig 2a-d, g, i; Fig 3b-g; Fig 4b-d, f-g, i; Fig 5d-f, Fig 6a-c, f-h; Fig 7c-h; Fig S1a, e-f; Fig S2a,c; Fig S3d-e; Fig S4d-g; Fig S5a-g; Fig S7a are available in the source data file.

Field-specific reporting

Please select the one below that is the best fit for your research. If you are not sure, read the appropriate sections before making your selection.

- [x] Life sciences
- [ ] Behavioural & social sciences
- [ ] Ecological, evolutionary & environmental sciences

For a reference copy of the document with all sections, see nature.com/documents/nr-reporting-summary-flat.pdf

Life sciences study design

All studies must disclose on these points even when the disclosure is negative.

Sample size	No sample size calculation was performed prior to experiments. At least three independent replicates were performed for each experiment in order to obtain statistical significant data. The precise number of replicates are described in the figure legends.
Data exclusions	In some microscopy experiments, some field of views are removed due to autofocus errors.
Replication	The reported results were replicable and the precise number of experimental repeats are described in the figure legends.
Randomization	Samples were not randomized as we use automated analysis tools to ensure an objective analysis of the data. Covariates are not relevant for the study as we always include a control that is treated similarly to the experimental sample.
Blinding	Blinding was not used in this manuscript as it was not found relevant for the applied experimental approaches. Blinding was not applied as quantitation for cell experiments was performed utilising automated image analysis tools (CellProfiler) to ensured the same criteria for object and intensity detection was being applied equally across experimental samples and negated bias.

Reporting for specific materials, systems and methods

We require information from authors about some types of materials, experimental systems and methods used in many studies. Here, indicate whether each material, system or method listed is relevant to your study. If you are not sure if a list item applies to your research, read the appropriate section before selecting a response.

Materials & experimental systems

n/a	Involved in the study	
	x	Antibodies
	x	Eukaryotic cell lines
x		Palaeontology and archaeology
x		Animals and other organisms
x		Human research participants
	x	Clinical data
x		Dual use research of concern

Methods

n/a	Involved in the study	
x		ChIP-seq
	x	Flow cytometry
x		MRI-based neuroimaging

Antibodies

Antibodies used

Primary antibodies (Concentrations used can be found in the methods section)

LC3B (Cell Signaling, #3868)
LC3B (MBL, #PM036)
p62 (BD-Biosciences, #610833)
GRAMD1C (Sigma, #HPA012316)
TOMM70A (Sigma, #SAB1401393)
Tubulin (Sigma, #T5168)
Actin (Cell Signaling, #3700)
EGFP (Takara, #632381)
mCherry (Thermo Fisher, #PA534974)
TOM20 (Santa Cruz, #17764)
ACSL4 (Santa Cruz, #SC-365230)
TIM23 (BD Bioscience, #611223)
COXIV (Cell Signaling, #4850)
ATG13 (Cell Signaling, #13468)
ATG16L1 (MBL, #PM040)
WIPI2 (Abcam, #Ab105459)
total OXPHOS antibody (Abcam, #ab110413)

	PDH (Cell Signaling, #2784s)<br>GAPDH (Cell Signaling, #5174)<br>p70S6K (Cell Signaling, #9202)<br>phospho-p70S6K (Cell Signaling, #9205)<br>MIRO2 (Proteintech, #PA5-52960)<br><br>Secondary antibodies:<br><br>DyLight 680 (Thermo Fisher, #SA5-10170)<br>DyLight 800 (Thermo Fisher, #SA5-10044)<br>Anti Rabbit Alexa Fluor 488 (Invitrogen, #A-21206)<br>Anti Mouse CY3 (Jackson, #115-165-146)
Validation	LC3B (Cell Signaling, #3868) - 1233x product citations on manufacturer's website(Validation Western blot+siRNA, IF+Chloroquine)<br>LC3B (MBL, #PM036) - 113x product citations on manufacturer's website(Validation Western blot + ATG5 KO)<br>p62 (BD-Biosciences, #610833) - 5x product citations on manufacturer's website (Validation Western blot)<br>GRAMD1C (Sigma, #HPA012316) - Prestige antibody validated by Human Protein Atlas (HPA) Project<br>TOMM70A (Sigma, #SAB1401493) - 7x product citations on manufacturer's website (Validation Western blot + TOMM70 overex)<br>Tubulin (Sigma, #T5168) - 3277x product citations on manufacturer's website (Validation western blot)<br>Actin (Cell Signaling, #3700) - 2650x product citations on manufacturer's website (Validation western blot)<br>EGFP (Takara, #632381) - 76x product citations on manufacturer's website (Validation western blot + AcGFP overex)<br>mCherry (Thermo Fisher, #PA534974) - 36x product citations on manufacturer's website (Validation western blot + mCherry overex)<br>TOM20 (Santa Cruz, #17764) - 464x product citations on manufacturer's website (Validation western blot, IF)<br>ACSL4 (Santa Cruz, #SC-365230) - 37x product citations on manufacturer's website (Validation western blot, IF)<br>TIM23 (BD Bioscience, #611223) - 27x product citations on manufacturer's website (Validation western blot)<br>COXIV (Cell Signaling, #4850) - 383x product citations on manufacturer's website(Validation western blot, IF, IHC + blocking peptide)<br>ATG13 (Cell Signaling, #13468) - 45x product citations on manufacturer's website (Validation western blot + overex ATG13)<br>ATG16L1 (MBL, #PM040) - 12x product citations on manufacturer's website (Validation western blot, IF)<br>WIPI2 (Abcam, #Ab105459) - 40x product citations on manufacturer's website (Validation western blot + Knockout)<br>total OXPHOS antibody (Abcam, #ab110413) - 841x product citations on manufacturer's website (Validation western blot)<br>PDH (Cell Signaling, #2784s) - 530x product citations on manufacturer's website (Validation western blot)<br>GAPDH (Cell Signaling, #5174) - 2501x product citations on manufacturer's website (Validation western blot)<br>p70S6K (Cell Signaling, #9202) - 1494x product citations on manufacturer's website (Validation western blot)<br>phospho-p70S6K (Cell Signaling, #9205) - 1409x product citations on manufacturer's website (Validation western blot)<br>MIRO2 (Proteintech, #PA5-52960) - 14x product citations on manufacturer's website. KD validated. (Validation western blot, IF, IHC)

Eukaryotic cell lines

Policy information about cell lines

Cell line source(s)	All cell lines (U2OS, HEK293T, 786-O and A498) obtained from ATCC
Authentication	All cell lines used were derived from ATCC authenticated cell lines
Mycoplasma contamination	All cell lines were regularly tested for mycoplasma contamination
Commonly misidentified lines (See ICLAC register)	No commonly misidentified cell lines according to the ICLAC register.

Clinical data

Policy information about clinical studies

All manuscripts should comply with the ICMJEguidelines for publication of clinical research and a completedCONSORT checklist must be included with all submissions.

Clinical trial registration	N/A
Study protocol	N/A
Data collection	N/A
Outcomes	N/A

Flow Cytometry

Plots

Confirm that:

☒ The axis labels state the marker and fluorochrome used (e.g. CD4-FITC).

☒ The axis scales are clearly visible. Include numbers along axes only for bottom left plot of group (a 'group' is an analysis of identical markers).

☐ All plots are contour plots with outliers or pseudocolor plots.

☒ A numerical value for number of cells or percentage (with statistics) is provided.

Methodology

Sample preparation	siRNA transfected U2OS wt cells were stained with CellRox (#C10422, Thermo Fischer) or Tetramethylrhodamine (TMRE) (#T669, Thermo Fisher) for 10 minutes according to manufacturer's instructions. After washing cells were trypsinised and washed in PBS twice.
Instrument	BD™ LSR II flow cytometer
Software	FlowJo
Cell population abundance	100,000 cells per sample were transfected and acquired 20,000 cells per sample were analysed in the single cell gated population.
Gating strategy	Live cells were first gated using FSC/SSC (gate 1). Cells from gate 1 were further gated using FSC-A/FSC-H for single cell separation (gate 2). 20,000 cells in gate 2 were analysed for CellRox or TMRE expression using FSC/Texas-red.

☒ Tick this box to confirm that a figure exemplifying the gating strategy is provided in the Supplementary Information.
